# A Model for the Remote Deployment, Update, and Safe Recovery for Commercial Sensor-Based IoT Systems

**DOI:** 10.3390/s20164393

**Published:** 2020-08-06

**Authors:** Alexandru Radovici, Ioana Culic, Daniel Rosner, Flavia Oprea

**Affiliations:** Computer Science Department, Automatic Control and Computer Science Faculty, University Politehnica of Bucharest, 060042 Bucharest, Romania; alexandru.radovici@upb.ro (A.R.); ioana_maria.culic@upb.ro (I.C.); flavia.oprea@upb.ro (F.O.)

**Keywords:** internet of things (IoT), application deployment, remote update, embedded software, sensor, embedded software update, software versioning

## Abstract

Internet of Things (IoT) systems deployments are becoming both ubiquitous and business critical in numerous business verticals, both for process automation and data-driven decision-making based on distributed sensors networks. Beneath the simplicity offered by these solutions, we usually find complex, multi-layer architectures—from hardware sensors up to data analytics systems. These rely heavily on software running on the on-location gateway devices designed to bridge the communication between the sensors and the cloud. This will generally require updates and improvements—raising deployment and maintenance challenges. Especially for large scale commercial solutions, a secure and fail-safe updating system becomes crucial for a successful IoT deployment. This paper explores the specific challenges for infrastructures dedicated to remote application deployment and management, addresses the management challenges related to IoT sensors systems, and proposes a mathematical model and a methodology for tackling this. To test the model’s efficiency, we implemented it as a software infrastructure system for complete commercial IoT products. As proof, we present the deployment of 100 smart soda dispensing machines in three locations. Each machine relies on sensors monitoring its status and on gateways controlling its behaviour, each receiving 133 different remote software updates through our solution. In addition, 80% of the machines ran non-interrupted for 250 days, with 20% failing due to external factors; out of the 80%, 30% experienced temporary update failures due to reduced hardware capabilities and the system successfully performed automatic rollback of the system, thus recovering in 100% of the temporary failures.

## 1. Introduction

The impact of the Internet of Things (IoT) based systems has grown in the past few years both in terms of device numbers and application fields, as well as in scientific and economic impact. IoT systems deployments are becoming ubiquitous, with dedicated solutions covering use-cases from agriculture, smart cities, or industrial applications, to medical wearable devices or smart and connected home-entertainment devices. Numerous businesses are using IoT based solutions both in various automatic facilities, and in large scale, IoT based distributed sensors networks deployments, used to gather real-time data for data-driven decision-making.

**Figure 1:**
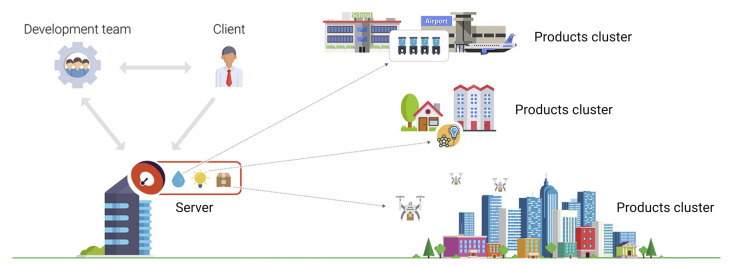
The IoT ecosystem.

Current advancements in IoT infrastructures lead to highly integrated solutions, empowering businesses to quickly deploy commercial solutions based on IoT devices while masking the complexity behind the multi-level implementation. However, behind the simplicity offered by these solutions, complex architectures are usually hidden [1], with numerous interconnected components that are required—from sensors, and hardware platforms, to embedded software, wireless communication infrastructures, special purpose gateways, networking solutions, cloud storage, and various data representation models, to final processing, data analytic tools, and custom view models. Figure 1 depicts the complexity and variety related to the IoT ecosystem.

All these complex systems rely on data collected or actions performed by the first level of the IoT implementations—the on-site hardware devices. These run custom software that is usually platform-dependent and has to answer to numerous constraints due to limited hardware capability and low power-consumption requirements. This software also requires updates and improvements, both for bug fixing, for new functionalities, as well as for security enhancements. Moreover, numerous IoT solutions are developed by fast-growing start-ups that lunch new versions frequently, thus requiring frequent software updates [2]. Finally, commercial applications usually have long life cycles for hardware components, needed to ensure proper amortisation—thus limiting system modification to software-only updates.

While software updates for embedded hardware have thus become a critical link in the IoT ecosystem, many industrial IoT deployments still lack the support for remote software updates—with all the updates being applied on location, through dedicated hardware interfaces. This generates high maintenance costs, as for each IoT device deployed in production, a technician needs to physically access it in order to launch and supervise the update process. On the other hand, plenty of companies have implemented dedicated Over The Air (OTA) update mechanisms, but many of these are far from supporting a secure and fail-safe application update process. Various cases of bricked or hacked devices as a result of a failed update have been reported [3,4]. This outlines an obvious need for a complex and secure remote update solution dedicated to IoT devices that covers all the layers in the IoT stack. Lately, platforms such as Android Things [5], Ubuntu Core [6], Mender [7], and Balena [8] (formerly called Resin) have been developed with the aim to support such fail-proof updates, targeting embedded devices.

In this context, this article aims to provide both an overview of current solutions for remote IoT software deployment, monitoring, and updating, as well as a novel model for tackling this challenge. To this end, we propose a generic mathematical model based on which various implementations of IoT deployment and update infrastructures, adapted to specific use-cases, can be built. Our model focuses on defining the key elements of a generic sensor-based application’s update infrastructure and the relationships between them. Further on, we have applied our model to a specific sensors system and we have built a medium scale IoT deployment refined through a considerable number of software updates.

In order to test the proposed model, we have implemented a remote software deployment system together with a commercial partner. This partner required 100 smart soda dispenser machines to run an embedded custom control and sensing software that undergoes frequent updates, both for bug fixing and for adding new functionalities. Based on our model, we implemented the software system that allows for the remote deployment, update, and safe recovery of the custom embedded software, for the 100 soda dispensers, distributed across three locations. Over a period of 250 days, 133 different patches and updates were remotely applied, with excellent results in terms of reliability.

The following work is structured as follows. Section 2 explores current adoption challenges specific to the IoT field with a focus on update systems for IoT applications together with an overview of available solutions that tackles the updates problem. Section 3 introduces our proposed model, with an in-depth view of the perceived design constraints and a mathematical model aimed at ensuring scaleability and robustness. Section 4 introduces the implemented experimental setup, while Section 5 describes how we conducted the tests and obtained results. The remaining section draws the main conclusions for this work.

## 2. State of the Art in Remote IoT Software Deployment

In terms of large scale commercial adoption, the Internet of Things is still in its early stages, as both numerous challenges, as well as practical business constraints are creating a gap between state of the art and real-world large scale deployments. While the advantages of integrating sensor-based IoT solutions in specific fields such as agriculture, health, smart cities, and even industrial facilities, are heavily outlined in both the research and the commercial literature [9,10], the actual adoption of these technologies is still far from reaching its full potential.

According to a 2017 Cisco survey carried out among over 1500 IT companies, 74% of surveyed organizations have failed with their IoT initiatives [11]. A number of surveys [12,13,14,15,16] or business reviews [17] have highlighted the reduced adoption of IoT technologies and the high percentage of reported failures for IoT deployments. The building consensus is that IoT systems complexity poses major challenges in terms of development, deployment, and platform management.

### 2.1. Specific Challenges Related to Deployment and Updates

IoT systems are designed as autonomous devices, so, in a simplistic manner, they can be described as mechanisms that retrieve environmental data and respond with specific actions. As they are widely integrated into dynamic use-cases that require the system to adapt to changing parameters, their software demands constant change. Even more, at the hardware level, the IoT infrastructures need to be expandable and to be able to easily integrate extra sensor nodes in the device network.

An important driver of required updates lies in the way commercial and industrial IoT applications are developed. As most companies adopt an agile methodology, the product requirements and specifications are under constant change. Therefore, many IoT products are released while still under development, as companies rely on pushing updates to add new product features and to improve the user experience.

Naturally, this comes in addition to the frequent firmware and software updates needed to ensure a proper response to the characteristic volatility related to IoT technologies, as well as the very high level of security expected of an IoT system. Independent of the employed technology, mitigating security risks is often described in direct relation with the need of updates that address the latest attacks and vulnerabilities [18,19].

While large-scale application deployment and updates are considered a significant factor in the commercial IoT development process, they are also looked upon as an important development challenge [20] and many of the commercial IoT devices currently available on the market lack any update mechanism [21]. We explore contributing factors next.

#### 2.1.1. The Heterogeneity of the IoT Ecosystem

**Figure 2:**
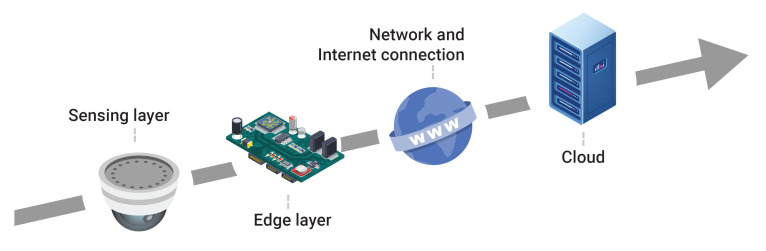
The IoT stack.

As the IoT evolved from simple sensors or actuators to complex networks designed to provide valuable insights for data-driven decisions, with multiple layers of abstractions, it becomes more and more complex. A typical IoT stack is depicted in Figure 2 starting from the sensing layer, where simple micro-controller based sensors and actuators are deployed, followed by the edge layer, where embedded computers are used to gather data and do primary processing tasks, and concluding with the cloud layer, where high-performance computers are employed with the purpose of large-scale data aggregation and analysis. For each level in this stack, a large variety of technologies is available, from different vendors and with different pros and cons. Furthermore, the total number of devices employed is very high in comparison with other areas such as smartphones or computers. A Gartner study predicts that the total number of connected devices by 2021 will be around 25 billion [22].

The Singh and Kapoor survey [23] offers an insight on some of the current hardware platforms available—presenting nine main variants. Of course, each manufacturer also offers numerous sub versions for its hardware platforms, further optimized for specific applications, with each sub version requiring various levels of customization to be integrated into an IoT infrastructure.

#### 2.1.2. Remote Device Diagnostic

Diagnosing a malfunctioning IoT device is a costly process. While for some gadgets that have a display in their configuration, such as smartwatches, users can report visible error messages, most of the devices are embedded within a larger system, and visible errors cannot be reported. Therefore, in the case of a failed or malfunctioning update, diagnosing the device and identifying the problem can represent a challenge and generate high costs. This is one of the key reasons why many manufacturers choose to reduce the number of software updates and minimize the risk of failed updates.

#### 2.1.3. Hardware Constraints

IoT systems are designed to integrate into the environment and become seamless technologies helping us achieve ambient intelligence. Therefore, most of the devices deployed need to integrate into existing everyday objects and run uninterrupted for long periods. This leads to specific constraints in the hardware employed. In general, IoT sensors and gateways are designed as small-size, low-energy consumption devices. This results in most of the hardware having reduced capabilities, in terms of processing power and available memory [24].

#### 2.1.4. Security

Security is considered one of the biggest concerns related to IoT, and it is mentioned in all research and commercial reports focused on IoT challenges and adoption issues [14,17]. In the IoT architecture, sensitive data collected by the endpoints are transmitted and processed at edge level and further on, stored in the cloud. Therefore, protecting this easily accessible data is of vital importance. Furthermore, many IoT devices are deployed to control machines such as heating/cooling systems, home appliances, medical devices, etc. Malicious control over these gadgets can peril human lives and result in catastrophic damages.

According to a Gartner 2019 report [25], security and privacy are the top two barriers for companies in achieving success in implementing IoT technologies and the overall lack of trust in a secure environment governed by ubiquitous IoT technologies is strongly related to the lack of sufficient updates [19,26].

In order to assure a high level of security and build certifiable IoT application update systems, specific strategies that make use of cryptographic algorithms [27], digital signatures, and execution policies are implemented [28] to handle both application-related deployments and kernel-related updates.

### 2.2. Existing Remote IoT Software Deployment Solutions

Research literature and commercial studies propose various models and implementations for over-the-air software deployment dedicated to constrained devices. Considering the variety of challenges related to this process, each proposed architecture focuses on a limited subset of the previously identified issues.

#### 2.2.1. Data Transmission-Focused Models

These implementations rely on efficient and secure data transmission for the remote update models.

Thantharate et al. [29] propose two over-the-air update solutions, one using MQTT and the other CoAP for data transmission, while Park et al. [30] propose a different CoAP-centered deployment system adapted for wireless sensor networks. Their two-phase model uses edge gateways to disseminate the data coming from the upper layer of the IoT stack and reduce traffic between the sensors and the external network.

#### 2.2.2. Firmware-Dedicated Solutions

Extensive research has been carried out in terms of firmware deployment mechanisms. Considering the extreme constraints related to low-energy and other specialized devices built around microcontrollers, several efficient and secure ways of over-the-air updates have been modeled and implemented.

Kerliu et al. [31] addressed the challenge related to the increased number of end nodes and implemented a solution meant to efficiently broadcast data into large sensor networks. With the same purpose, UpKit [32] is an end-to-end deployment infrastructure meant to cover all phases of the update process from update firmware generation to data transmission, packet verification, and flashing on the device.

Other firmware update solutions are modeled based on the challenges related to the specific implementation field such as automotive [33], smart cities [34], or wearables [35].

#### 2.2.3. Software-Dedicated Solutions

When targeting embedded computers, the implementations are more general when compared with the previously presented solutions. This is mainly because the hardware layer withstands tasks that are more processing and memory consuming.

In this context, ThingsStore [36] proposes a marketplace that aggregates devices and applications that interact via event queries. The platform aims to abstract the heterogeneity of the hardware layer and acts as a hub for three main actors: devices, applications, and users.

Udoh and Kotonya [26] made a review of other existing IoT development tools. Out of the eight different platforms analyzed, only three implement deployment and maintenance mechanisms: D-LITe [37], IoTSuite [38], and RapIoT [39]. However, the review outlined that the emphasis is placed on the development process rather than on the software updates.

#### 2.2.4. Commercial OTA Update Solutions

Software and firmware deployments and updates are essential for any IoT solution, but the overview of existing solutions described above denotes an obvious lack of mature, commercially usable platforms. This is also because many producers have implemented custom OTA updates systems designed to integrate with their technologies [40,41]. However, many of these systems are poorly researched and implemented, exposing the IoT products to security risks and important failures.

A famous example of a software update gone bad is related to the LockState smart locks which are widely used in Airbnb homes. In 2017, an OTA firmware update made the built-in keypad nonfunctional as the devices got locked out of the company’s servers, making another wireless update impossible [3]. In the automotive industry, Mecedes was affected by a failed update that exposed the car owner’s information to other users [4].

The aforementioned firmware and software update solutions, extracted from the research literature, are still under development and have not been integrated into commercial use-cases. They are theoretical models tacking specific challenges but are not designed for commercial and industrial use.

On the other hand, companies such as Google and Canonical have developed IoT update systems targeting constrained devices, which have been integrated into various use-cases. The main existing solutions for OTA updates are Android Things [5], Ubuntu Core [6], Mender [7], and Balena [8] (Table 1).

Android Things [5] is a full-stack software development and deployment solution developed by Google based on the Android framework. The process of building, deploying, and updating IoT applications using the Android Things platform is similar to the development of Android smartphone applications and requires an Android Console account.

Ubuntu Core [6] is an IoT platform based on the Ubuntu Linux [42] distribution that uses the snap package manager to enable the deployment of applications on IoT devices. Similarly, Mender [7] is an open source IoT application development and deployment system based on Yocto Linux [43]. For both Mender and Android Things, updates are made in a robust manner using a dual partition mechanism.

Similar to Mender, Balena [8] uses Yocto Linux distributions to implement software deployment and update mechanisms for IoT devices. The main difference between the two lies in the implementation, as Balena uses docker containers [44] for application deployment.

### 2.3. Conclusions on Existing OTA Update Solutions

Considering the OTA updates solutions analyzed above, we have identified that the research literature lacks mature solutions that can be implemented into commercial and industrial use-cases. While many platforms dedicated to secure software and firmware updates are being researched, the architectures are not completely implemented and lack the specific use-cases. Furthermore, most of these solutions focus on efficient data transmission and integrity checks, but lack process isolation mechanisms that are equally important in ensuring the security of the entire IoT platform.

On the other hand, some commercial solutions that are still undergoing improvements have been developed by IoT companies, and many of them are developed to address specific use-cases and are built on top of proprietary cloud technologies, forcing the vendors to integrate their products with specific cloud platforms.

In this context, we identified a gap related to IoT deployment and update solutions that are built to address market needs using open technologies, while also modeled on top of a strong theoretical foundation. To this end, our aim is to propose a solution which is endorsed by a generic mathematical model, while also suitable for specific commercial implementations on the vendors’ premises.

## 3. Proposed Model

### 3.1. General Characteristics of IoT Update Systems for Commercial Applications

Based on the above-mentioned challenges and concerns affecting the development, and maintenance of commercial and industrial IoT technologies, we have defined specific characteristics we consider essential for an effective IoT deployment and updates system targeting commercial applications. In the case of commercial products, usually the update is delivered on a functioning platform that is either in use on the customers’ premises or as a component of a larger network of sensors and gateway devices as the purpose of the deployment is to deliver new features, and fix bugs, or security breaches [45]. Therefore, we propose the following characteristics for an update system designed to deploy applications onto varied commercial hardware devices.

*Remote Location*
  Systems integrated with fields such as agriculture, mobility, and weather monitoring are usually difficult to reach, making any update process that requires a physical connection to the device highly resource consuming. Therefore, a key characteristic of the deployment system we aim to model is to support the remote deployment of updates so data to be flashed on the hardware platform can be transferred over the air. Such a system can leverage the connectivity characteristic to all IoT platforms and integrate with the existing infrastructure on top of which data transfers can be made.
*Transactional*
  The software updates performed on the end-nodes and gateways should be made in a transactional manner to prevent device failures related to network connectivity issues (e.g., the Internet connection gets suspended) or faulty data write. In this case of an interrupted or faulty deployment process, the update changes are not committed and the device continues to run the previous software version. This way we can preserve the equipment’s functioning in case of an update failure.
*Differential Updates*
  Another characteristic meant to address unstable and limited connectivity issues is to implement differential updates. By storing distinct software versions in a differential manner, only the changes between the latest and the preceding software versions will be transferred. This results in significantly fewer data to be transmitted to the devices, compared to the case when an entire application or an application bundle is uploaded onto the network. While this increases the complexity of the deployment system, it preserves bandwidth which in some cases can be limited.
*Versioning*
  Updates pushed on commercial products need to be carefully tracked for several reasons. This is why versioning is used for any software or hardware equipment available on the market. Any update management platform needs to be designed so versions can be easily recorded and managed by the development team.
*Rollback*
  This feature is described in close relation with the previously mentioned characteristic, versioning the software updates. Once an effective versioning model is in place, it needs to be integrated with a rollback system that can change the software version running on the IoT infrastructure. This feature is essential in case major issues related to the software are identified or certain hardware equipment is not compatible with the newly deployed software [46]. In such an unfortunate situation, the application can be switched with a previous version for all or for a specific class of devices.
*Device Lockout and Bricking*
  A poorly implemented update can result in the lockout or bricking of the IoT devices [40]. This makes the product unusable and generates high maintenance costs for the vendor, who will need to physically access the device for repair or choose to exchange the product. In our deployment system model, we carefully tackle this issue and design an update method that prevents any situation that can result in a lockout.
*Isolation*
  In modeling the deployment platform, we find it important to create a modular system where each component runs in a dedicated sandbox. This isolation ensures the device can preserve partial functionality albeit some of the applications might not be working properly. As long as the malfunctioning software component does not impact equipment connectivity and the deployment infrastructure, remote diagnosis and additional updates can be made entirely remotely.
  Furthermore, application isolation also brings advantages from the security point of view. In the unfortunate case of an attack compromising one of the software components, the rest of the system is not affected.
*Security Layer*
  Another key aspect we need to consider in the deployment system model is security, more specifically, we refer to the data transmitted during the deployment process. For remote updates, the new software version been transmitted is exposed to various data transit attacks. Therefore, a security layer protecting all the elements in the sensors and gateways network is required. This needs to implement several security policies capable of authenticating and authorizing the source and making an integrity check to certify that all the data reaching the end-nodes and gateways have not been tampered with [46].

### 3.2. Proposed Development and Deployment Model

In this paper, we propose a system development and deployment model that may be used in commercial applications. The model aims to aggregate the advantages of all the above-described technologies to enable robust and fail-safe application updates for complex IoT systems.

In comparison with other existing solutions, our approach aims to stand out as a generic model that addresses the main aspects related to commercial IoT software deployment and updates in a non-specific manner. Therefore, we start by defining the general requirements as a mathematical model that can be implemented according to specific use-cases. Furthermore, we offer a technical implementation with a twofold purpose: to validate the mathematical model and to offer an open platform that can be easily integrated and adapted to specific use-cases. From this point of view, the proposed solution differs from the other existing commercial platforms by being more generic and by being entirely developed on top of open technologies without forcing the integrators to comply with specific frameworks. In addition, by being generic, our implementation addresses all the software update requirements identified in both research and commercial literature.

#### 3.2.1. Design Constraints

In the development of the model, we started from the following constraints:

**Design Constraint 1.** *There is a need to develop and debug applications on real hardware using an environment as close as possible to the real operating conditions. The software may be developed in the laboratory, but migrating towards the real hardware and deployment environment is difficult.*

**Design Constraint 2.** *After the software development and the debugging stages are complete, there is a need for beta testing on the actual devices in the real functioning environment. These devices should be identical to the production devices and located in an environment having similar parameters to the one where they are designed to be deployed in production.*

**Design Constraint 3.** *Once the development and testing are finalized, the application updates have to be deployed incrementally, so that errors in the deployment process can be spotted early on.*

**Design Constraint 4.** *There is the need for scheduled software deployments so that production devices do not update during operation times (e.g., do not update a coffee machine while brewing coffee or a vending machine while it performs a sale).*

**Design Constraint 5.** *If the new software version does not start on specific equipment, it needs to be automatically rolled back to a safe version bringing the device to the exact state as before the update.*

**Design Constraint 6.** *Operators need to have access to a central dashboard where they can monitor the devices and their behavior, run diagnostics tests, repair the devices remotely, and manually rollback the software on certain categories of devices.*

**Design Constraint 7.** *Device owners have to be able to disable any managed devices.*

**Design Constraint 8.** *Devices need to have some way of authentication and be compatible with third-party Trusted Platform Modules (TPM) [47], such as ARM TrustZone [48] or Software Guard Extensions [49].*

**Design Constraint 9.** *Updates should be as fast as possible and require as little as possible bandwidth.*

**Design Constraint 10.** *The system should be able to integrate with whatever architecture the vendor has.*

To this end, we propose a system model designed to enable the development and remote deployment of software applications on embedded computers, based on the premises we identified above.

#### 3.2.2. Proposed Terminology

The proposed model relies on a central unit orchestrating all the connections, deployment procedures, and device management related operations. In this process, the system is built upon the following key objects: Vendor, User, Product, Cluster, Application, Container, Deployment, Project, and Event, which are used in relation to other actors. Further on, we detail the terminology used to describe the model.

- Vendor—The vendor is the entity that uses the system to build and manage hardware devices. In other words, the vendor is the IoT solution producer, which in this case we identify as the user of the system. Each vendor owns projects, clusters, products, applications, deployments, and containers.
- User—Each vendor may define several users that are allowed to manage objects (described below).
- Product—A product is a single device. We define it as a product as it is the actual item that is being sold by the vendor. Each product has a unique id and a name (that may not be unique) and is part of a cluster. There are three types of products: development—products that are used during development and debugging; beta-products that are used for beta testing; production—products sold by the vendor.
- Cluster—A cluster is a group of products (devices). They usually run the same software and are located in one geographical area (e.g., a collection of sensors and gateways aggregating temperature information).
- Application—An application is a piece of information uniquely identifying the software that will be deployed to a cluster or a product. Each application has a list of available version numbers and a set of default parameters used to run the deployed piece of software.
- Container—This is the actual software package deployed to a device. It is stored in a repository.
- Deployment—A deployment is a link between a target (may it be a cluster or a product), an application, a specific application version number, and a set of run parameters. After creating a deployment, all target products will make sure that they run the latest version of that application. When deleting a deployment, all targets will make sure they rollback the application to the previous version.
- Event—An event is a log of an action that has happened at a certain point in the infrastructure. Examples of events are login and logout of users, and product updates.

The software update system we propose relies on three main components: the products, the server, and the deployments, where the products interact with the server and run applications packaged as deployments. To describe this complete infrastructure, we have developed a mathematical model that aims to serve as an abstraction for a generic IoT application update solution. Based on it, various systems addressing particular scenarios and use-cases can be implemented.

To describe the model we propose, we first have to define the following sets that will be used throughout this section:

- *P* the set of all possible product ids, the actual value types being defined by each technical implementation;
- *C* the set of all possible cluster ids, the actual value types being defined by each technical implementation;
- *K* the set of all possible public key infrastructure (PKI), keys, such as RSA [50] or ECC [51];
- $P_{a}$ the set of all possible additional parameters, the type of values from this set will be defined by each technical implementation;
- *A* the set of all possible application ids, the actual value types being defined by each technical implementation;
- *S* the set of all possible digital signatures resulted from using the keys from *K*;
- *U* a set of unique tokens, used by a product for authentication purposes, the actual value types being defined by each technical implementation;
- *E* a set of errors that can appear, the actual value types being defined by each technical implementation.

Additionally, we define the set *T* consisting of the product types (1) available. We have defined three types of products: *development*, used for interactive application development and testing, *beta* used for testing applications in an environment similar to the production one, and *production* that are the actual devices deployed in the field:

(1) T={development,beta,production}

Using the sets defined above, we have developed a mathematical model defining the key components of the proposed IoT update infrastructure.

### 3.3. The Mathematical Model

In the proposed model, the product represents an abstraction of a device. Therefore, we define the space *M* (2) representing all the products. The product vector’s *dimensions* are its id, its cluster’s id, its product and cluster private keys, its type, and some additional parameters that are dependent on each technical implementation:

(2) $$M=P\times C\times K\times K\times T\times P_{a}$$

A product is represented by a vector $\vec{m}$ (3), also called *the manifest*:

(3) $$\vec{m}:M=\left(id_{product},id_{cluster},key_{cluster},key_{product},type\right)$$

We define the following projection functions for *M* vector space that allows us to obtain the vector components on each axis: the product function (4) projects $\vec{m}$ onto the $id_{product}\in P$, a value uniquely defining a product; the cluster function (5) projects $\vec{m}$ onto the $id_{cluster}\in C$, a value that uniquely identifies the cluster to which the product belongs to; the $k_{c}$ (6) and $k_{p}$ functions (7) project $\vec{m}$ onto the $key_{product}\in K$ and $key_{cluster}\in K$ representing the product’s private key and the cluster’s private key; the type function (8) projects $\vec{m}$ onto the type∈T representing the product type, and the parameters function (9) projects $\vec{m}$ onto the $parameters\in P_{a}$ representing other parameters that are specific to the implementation:

(4) $$product\left(\vec{m}\right):M\to P=\vec{m}\times {\left(1,0,0,0,0,0\right)}^{T}$$

(5) $$cluster\left(\vec{m}\right):M\to C=\vec{m}\times {\left(0,1,0,0,0,0\right)}^{T}$$

(6) $$k_{c}\left(\vec{m}\right):M\to K=\vec{m}\times {\left(0,0,1,0,0,0\right)}^{T}$$

(7) $$k_{p}\left(\vec{m}\right):M\to K=\vec{m}\times {\left(0,0,0,1,0,0\right)}^{T}$$

(8) $$type\left(\vec{m}\right):M\to T=\vec{m}\times {\left(0,0,0,0,1,0\right)}^{T}$$

(9) $$parameters\left(\vec{m}\right):M\to P_{a}=\vec{m}\times {\left(0,0,0,0,0,1\right)}^{T}$$

We define the $k^{T}$ the public key corresponding to the private key k∈K.

#### 3.3.1. The Deployment Model

A deployment is any version of an application that can be run on one or multiple products. The deployment is built based on containers, which are the actual elements than get deployed and run on the products. In this model, we define the set of all available containers as a repository (*R* (10)). An element of this set identifies an application and ties it to one of its versions.

(10) R⊂A×N

To represent all the versions of an application, we define a function *v* (11).

(11) $$v\left(id_{app}\right):A\to P\left(N\right)=\left\{n|(id_{app},n)\in R\right\}$$

**Remark** **1.** *As outlined in (11), an application can be defined without having any versions. This means that there is no code that can be run for that application at the current moment.*

Further on, we define two types of deployments: cluster-bound deployments and product-bound deployments. A cluster-bound deployment is a mapping between a container of *R*, a cluster of *C*, and a product type of *T* (12).

(12) $$D_{C}\subset R\times C\times T$$

A product-bound deployment is a mapping between a container of *R*, a product of *P*, and a product type of *T* (13):

(13) $$D_{P}\subset R\times P\times T$$

The list of all containers that have to exist on a product that is part of a cluster-bound deployment is obtained by using the $d_{c}$ function (14). These containers reside on the product’s storage but are not necessarily run.

(14) $$d_{c}\left(\vec{m}\right):M\to P\left(R\right)=\left\{\left(id_{app},v_{app}\right)|(id_{app},v_{app},cluster\left(\vec{m}\right),type\left(\vec{m}\right)\in D_{C}\right\}$$

The list of all containers that have to exist on a product in the case of a product-bound deployment is obtained by using the $d_{p}$ function (15). These containers reside on the product’s storage but are not necessarily run:

(15) $$d_{p}\left(\vec{m}\right):M\to P\left(R\right)=\left\{\left(id_{app},v_{app}\right)|(id_{app},v_{app},product\left(\vec{m}\right),type\left(\vec{m}\right)\in D_{P}\right\}$$

**Remark** **2.** *From (14) and (15), we can infer that a product might store several versions of the same application.*

We define the function *d* (16) as the union between the set of containers that have to be stored on a product that is part of a cluster-bound deployment and the set of containers that have to be stored on a product that is part of a product-bound deployment:

(16) $$d\left(\vec{m}\right):M\to P\left(R\right)=d_{c}\left(\vec{m}\right)\cup d_{p}\left(\vec{m}\right)$$

**Remark** **3.** *From (16), we can infer that a product might store zero or more containers.*

Another aspect we consider important in the modelling of this system is the containment of crashing applications. In this regard, we define $t_{crashes}$ as being a threshold of the number of times an application is allowed to crash (stop with an error) before the system marks it as non functional. This threshold is necessary as applications might crash for several reasons, some of them not related to the application itself. We define run (17) as the function that runs a version of an application using a product at a time *t*. The function returns the application’s exit error code. If this error code is 0, the application has exited successfully. Otherwise, the application is considered to have crashed:

(17) $$run(\vec{m},id_{app},v_{app},t):M\times R\times N\to Z$$

We also define the number of crashes (18) as a function dependent on each product, applications, and version, and it returns the number of times the run function has returned a value different from 0 in the $t_{o}-t$ interval:

(18) $$crashes\left(\vec{m},id_{app},v_{app},t\right):M\times R\times N\to N=\sum_{n=0}^{t}\left(run\left(\vec{m},id_{app},v_{app},n\right)\neq 0\right)$$

The containers that have to be run at a time *t* are defined by the function $r_{set}$ (19). The function takes as arguments the product manifest $\vec{m}$ and the time *t* and provides a set of *R* elements. Each element of *R* is uniquely identified by the application id which is linked to the highest version that is known to run without having crashed more than $t_{c}rashes$ times (the crash threshold). This is in agreement with design constraint 5, which states that applications should be rolled back to the latest stable version:

(19) $$r_{set}\left(\vec{m},t\right):M\times N\to P\left(R\right)=\left\{\left(id_{app},v_{app}\right)|\max\left(\left\{v_{app}|crashes\left(\vec{m},id_{app},v_{app},t\right)<t_{crashes}\right\}\right)\right\}$$

#### 3.3.2. The Server Model

The server is the component orchestrating the entire system. The server manages the application deployments and communicates with the connected products to deploy the new software version. Before describing the manner in which the product and the server exchange messages, we have to define the $req_{s}$ function.

$req_{s}$ (20) is a function that depends on the time variable *t* and it returns its value at the time t−1 increased by one unit:

(20) $$req_{s}\left(t\right):N\to N=req_{s}\left(t-1\right)+1$$

An exchange represents a pair of packets $\left(p_{req}\left(\vec{m},n,t\right),p_{res}\left(\vec{m},n,t\right)\right)$ defined by the functions (27) and (38) that are exchanged between a product $\vec{m}$ and the server at specific time intervals. Before we go into the details of an exchange, we have to define the specific elements involved.

#### 3.3.3. Token Generation

We have defined above the token as a unique element used for the authentication of the product. A new token element is generated by the token (21) function, which takes the time *t* and the product vector *m* as arguments:

(21) $$token\left(t,\vec{m}\right):N\times M\to N=\left\{\begin{array}{cc} t_{n}, & t\neq 0\wedge \forall t_{o}\in N,\forall m_{o}\in M,t_{n}\neq token\left(t_{o},\vec{m_{o}}\right)\wedge t_{o}\neq t\\ 0, & t=0 \end{array}\right.$$

#### 3.3.4. Request/Response Data

We design the communication between the server and the product as request–response pairs, based on the server–client paradigm. In this context, we define $D_{req}$ as the set of all possible request data that a product may send to the server and $D_{res}$ as the set of all possible response data that the server may send in reply. The actual request and response values are defined by each technical implementation.

The payload function (22) will collect and provide all the request data $req_{n}$ generated for the server since the last successful exchange:

(22) $$payload\left(\vec{m},n,t\right):M\times N\times N\to P\left(D_{req}\right)=\overset{t}{\underset{t_{r}\in Q=t-1}{⋃}}\left\{req_{n}\left(t_{r}\right)\right\}$$

We define the $P_{req}$ (23) set containing all the possible exchange packets that can be sent from the product to the server and the $P_{res}$ set containing all the possible exchange packets that can be sent from the server to the product:

(23) $$P_{req}\subset P\times N\times N\times U\times P\left(D_{req}\right)$$

#### 3.3.5. Nonce

We define nonce as a function (24) that receives a natural sequence number as a parameter and returns a unique number:

(24) $$nonce\left(n,t\right):N\times N\to N=u_{n},\forall k\in N,\forall t_{o}\in N\wedge k\neq n\wedge t_{o}\neq t\wedge nonce\left(k,t_{o}\right)\neq u_{n}$$

The nonce element is often used in the context of data-transmission security [52]. It is a cryptography element that is uniquely generated in a non-predictable way, usually using random number generators, for each transmitted packet to ensure that the same packet is not reused, thus preventing replay attacks [53]. As many products will report telemetry data whose content might be predictable, adding a nonce to the packets adds some randomness to it, thus making key inference harder. In a similar manner, we use the nonce function to make sure duplicate packets are not processed. Therefore, the server relies on a nonces (25) function that keeps track of the nonce numbers that have been received from each product before the time *t*:

(25) $$nonces_{p}\left(\vec{m},t\right):M\times N\times N\to P\left(N\right)=nonces_{p}\left(\vec{m},t-1\right)\cup req_{nonce}\left(p_{req}\left(\vec{m},t\right)\right)$$

We also define a $nonces_{s}$ (26) function that keeps track of the nonce numbers received by a product from the server:

(26) $$nonces_{s}\left(t\right):N\to N^{n}=nonces_{s}\left(t-1\right)\cup nonce_{s}\left(t\right)$$

#### 3.3.6. Sending/Receiving Exchange Packets

The communication between the server and the products is based on exchanges, which we defined above as pairs of request and response packets.

All the request packets are sent sequentially, each of them having a sequence number *n* as attribute. The function $p_{req}$ (27) describes one request packet. The result of the function contains the id of the product, a nonce, the sequence number *n*, the token provided by the server during the connect request, and the payload:

(27) $$p_{req}\left(\vec{m},n,t\right):M\times N\times N\to P_{req}=\left(product\left(\vec{m}\right),nonce\left(n,t\right),n,token\left(t,\vec{m}\right),payload\left(n\right)\right)$$

The final exchange packet is digitally signed using the $k_{p}\left(\vec{m}\right)$ as the signature is added to the request packet (28), and sent to the server:

(28) $$exchange_{req}\left(p_{req}\left(\vec{m},n,t\right)\right):P_{req}N\times N\to P_{req}\times S=\left(p_{req}\left(\vec{m},n,t\right),sign\left(p_{req}\left(\vec{m},n,t\right),k_{p}\left(\vec{m}\right)\right)\right)$$

Further on, we define the $req_{p}$ function (29) that projects the product id from an exchange packet. In a similar manner, (30)–(32) project the token, nonce, and sequence number *n* from a packet:

(29) $$req_{p}\left(p\right):P_{req}\to M=\vec{m},p\times {\left(1,0,0,.....\right)}^{T}=product\left(\vec{m}\right)$$

(30) $$req_{token}\left(p\right):P_{req}\to N=p\times {\left(0,0,0,1,.....\right)}^{T}$$

(31) $$req_{nonce}\left(p\right):P_{req}\to N=p\times {\left(0,0,1,0,.....\right)}^{T}$$

(32) $$req_{n}\left(p\right):P_{req}\to N=p\times {\left(0,1,0,0,.....\right)}^{T}$$

We also define the $req_{payload}$ function (33) that returns the payload vector associated with an exchange packet:

(33) $$req_{payload}\left(p\right):P_{req}\to N=p\times {\left(0,0,0,0,1,1,1,1,1,.....\right)}^{T}$$

When received by the server, the packet’s digital signature is checked for authenticity using the accept (34) function. If the check is successful, the packet is verified against packet replay [54] using the nonce and sequence number. If the exchange packet is accepted by the server, it uses the response function (35), to generate a response packet payload.

We define the $accept_{p}$ function (34) that the server applies to each received exchange packet to determine if a packet should be accepted or not:

(34) acceptp(p,s,t):Preq×S×N→B=1,signp,kpreqp(p)T=s∧reqtoken(p)=tokent,reqp(p)∧reqnonce(p)∉noncespreqp(p),t−1 0,otherwise

If the packet is accepted by the server, the server will process it and generate a response (35):

(35) $$response\left(\vec{m},n,t,req_{payload}\left(p\right)\right):M\times N\times N\times P\left(D_{req}\right)\to D_{res}=\left\{res_{1},res_{2},\ldots \right\},$$

We define the response packet vector space, containing all the possible packets sent from the server to the product, as $P_{res}$ (36):

(36) $$P_{res}\subset P\times N\times N\times \left(P\left(D_{res}\right)\cup E\right)$$

The function $p_{res}$ (38) describes one response packet. The result of the function contains the id of the product that the response is targeted at and the actual response (37):

(37) $$p_{res}:P_{req}\times K\times N\to P_{res}$$

(38) $$p_{res}\left(p,s,t\right)=\left\{\begin{array}{cc} \left(req_{p}\left(p\right),nonce_{s}\left(t\right),n,response\left(req_{p}\left(p\right),req_{n}\left(n\right),t,req_{payload}\left(p\right)\right)\right), & accept_{p}\left(p,s,t\right)=1\\ \left(req_{p}\left(p\right),nonce_{s}\left(t\right),n,error\in E\right), & accept_{p}\left(p,s,t\right)=0 \end{array}\right.$$

The exchange packet sent to the product is generated using the $exchange_{res}$ function (39). This takes as an argument the response packet (38) and the server key, signs the packet, and attaches the key:

(39) $$exchange_{res}\left(p_{res}\left(p,s\right)\right):P_{res}\times S\to P_{res}\times S=\left(p_{res}\left(p,s\right),sign\left(p_{res}\left(p\right),k_{s}\right)\right)$$

On the product side, when a packet is received, the accept function verifies the packet’s authenticity using the digital signature and whether it is a retransmitted packet using the nonce element:

(40) $$accept_{s}(p,s,t):P_{res}\times S\times N\to B=\left\{\begin{array}{cc} 1, & sign\left(p,k_{s}^{T}\right)=s\wedge \\ \; & nonce\left(p\right)\notin nonces_{s}\left(t-1\right)\\ 0, & otherwise \end{array}\right.$$

If the packet is accepted, the response data are sent to the product software components that will process the data. An example of possible data is the deployment set.

#### 3.3.7. Product Registration

Before a product can exchange packets with the server, it has to register to it. For this, the server stores a set of known products $P_{m}\subset M$, called *manually provisioned products*. Additionally, the server stores a set of known products $P_{s}\subset M$, called *self-provisioned products*. The union of the two sets defines all the registered products ($P_{all}=P_{m}\cup P_{s}\subset M$).

A product will use the $next_{p}$ function (41) to determine the next packet’s sequence number. The function relies on the result of the previous response. If this is an error, the function value is 0, which means the device has to register with the server before it can send any exchange packets:

(41) $$next_{p}\left(\vec{m},n,t\right):M\times N\times N\to N=\left\{\begin{array}{cc} 0, & response\left(p_{res}\left(p,s,n,t\right)\right)\in E\\ n+1, & otherwise \end{array}\right.$$

Depending on the provisioning type, the product will generate a product private key used by the $register_{s}$ function (42) to self-register or by the $register_{m}$ function (43) for a manually provisioned registration.

Each of the register messages is composed out of the product vector *m*, a nonce value, and a digital signature.

(42) $$register_{s}\left(\vec{m},t\right):M\times N\to M\times N\times S=\left(\vec{m},nonce\left(t\right),sign\left(\vec{m},nonce\left(t\right),k_{c}\left(\vec{m}\right)\right)\right)$$

(43) $$register_{m}\left(\vec{m},t\right):M\times N\to M\times N\times S=\left(\vec{m},nonce\left(t\right),sign\left(\vec{m},nonce\left(t\right),k_{p}\left(\vec{m}\right)\right)\right)$$

On the server side, the register message is authenticated and verified against packet replay by applying the $accept_{register}$ (44) function. If the packet is accepted, the server resets the packet counter *n* and generates a regular response packet that contains a product token:

(44) acceptregister(m→,s):M×N×S→B=1,(signm→,kp(m→)T=s∧nonce(p)∉noncesregister(t)∧product(m→)∈Pm)∪signm→,kc(cluster(m→))T=m→∧nonce∉noncesregister 0,otherwise

(45) $$register_{r}\left(\vec{m}\right):M\to M\times N\times S=\left(\vec{m},nonce,token,sign\left(\left(\vec{m},nonce,token\right),k_{s}\right)\right)$$

In this section, we have presented a generic mathematical model for a remote deployment system. This model can be applied in practice to any type of devices, from constrained devices to devices with more processing power. It provides a theoretical starting point of any remote deployment system. Most of the existing commercial deployment systems, such as Balena [8] or Mender [7] can apply to this model.

## 4. Test Implementation for Model Validation

Using the mathematical model described above, we have designed a reference implementation called IoTWay [55]. Our main focus, here, lies on using open standards and protocols that are proven to be safe and secure. Further on, we will describe all the components that we have implemented and how they relate to the mathematical model.

### 4.1. Proposed Architecture

**Figure 3:**
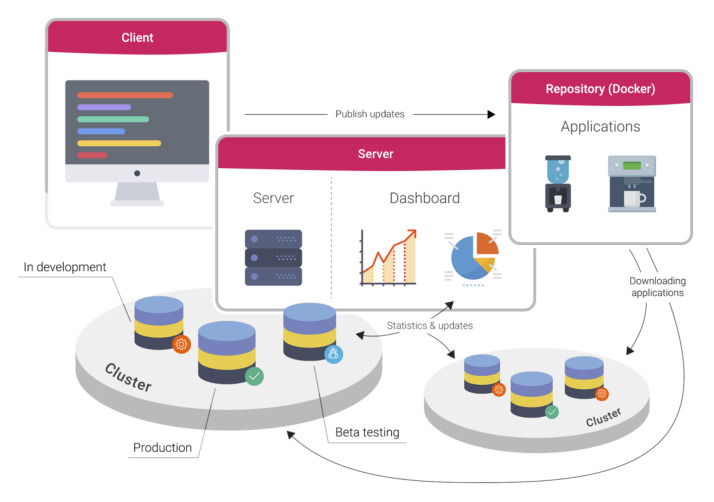
IoTWay system high-level architecture.

Our implementation has four main functional components, which we detail below: the server, the repository, the deployer, and the client. These components are depicted in Figure 3.

*The server* handles user and product authentication, cluster, product, application and deployment management, and event logging. As defined by the design, constraint 10 states that IoTWay should be able to integrate with any existing environment, the server is designed as a collection of web services accessed via a REST API interface. Data transfer is done using the HTTPS protocol with data in JSON [56] format.

*The repository* is a private air-gaped container repository. It relies on the server for authentication using a token bearer OAuth [57] method.

*The deployer* is a piece of software running on each product. It manages the product by handling container installation and running, file system mappings for the containers, and event logging. Optionally, the deployer offers an active (bidirectional) link between the product and the server used for a shell or a remote connection. This will be described further on.

*The client* is a pseudo component that allows vendors to interact with the server and the products. This component is optional, as vendors may choose to directly integrate the IoTWay server into their existing environment via JSON REST API.

The update mechanism starts with the deployer querying the server for the list of deployments (application and version) that are scheduled to run on the product. The server authenticates the product and provides the product deployer a list of deployments together with the set of credentials associated with the container repository. The deployer downloads the containers from the repository and manages them.

### 4.2. Details on the Server

The server is the central information and orchestration point; it is the component that keeps track of all the products, clusters, applications, deployments, users, and the associated access rights. It has several components: the user manager, the product manager, the application manager, the events manager, and the remote manager.

The user manager is responsible for user authentication. All of the objects, clusters, products, applications, deployments, and events belong to a user.

#### 4.2.1. User

The user is an object that represents an actual person that uses the system. Users are able to manage clusters, provision and manage products, define applications, manage deployments, view events, and interact with products using a remote connection.

A user is identified by a universal unique identifier (UUID). This enables users to be ported from one system to the other. A user owns clusters, products, applications, deployments, and projects.

#### 4.2.2. Cluster

A cluster is a grouping of products. Usually, products in a cluster are similar and run the same software. Similarly to users, clusters are identified by a UUID. A cluster has a name, a PKI key pair, and a list of allowed products (the only products that can connect to the server).

All products in a cluster must run on the same hardware and operating system. This is what we call the cluster’s platform.

The cluster also defines the way its products are provisioned: manually or self-provisioning. The first approach implies that the products are provisioned by the user. This can be done via a REST API call, so vendors might be able to integrate this into their systems. Usually, this method is used for development and beta products, as there are only a few of them. For production products, vendors have the option to provision them manually, via the REST API, or use the self-provisioning option. For the latter, each cluster uses a PKI key pair. Section 4.4.1 will discuss in detail the product authentication and provisioning.

As products can use different hardware and software platforms, there is no specific way to define how some actions should be performed. This cluster implementation allows the user to define several scripts that will be run on the products so the platform can be adapted to the user’s use case.

#### 4.2.3. Product

The product stores information about a device. Each device is identified by a UUID.

This model allows users to follow the complete product development life cycle. As defined by (1), there are three types of products:

- *Development* products have a special deployer installed on them, allowing developers to directly access the product by using a console and run applications on it. Applications are bundled into containers and deployed on the products, thus simulating an environment similar to the one in production.
- *Beta* products are identical in all aspects to the products deployed in the field. The only difference is a flag that tags them as beta devices. When deploying a new application or new application version, this is initially deployed onto beta products for testing purposes. When testing is done, deployments are upgraded into production.
- *Production* products are the ones deployed in the field. These are the products that the customers interact with.

From the security point of view, each product has a PKI key pair, an access token, and a symmetric key. The first two are used for product authentication as described by (28) and (42), while the symmetric keys are used for constrained hardware products authentication (where PKI digital signatures are not available due to technical limitations).

The implementation defines the following format for the Pa set:

- *serial*—a vendor defined string that uniquely identifies a product;
- *hardware*—a string defining the product’s hardware;
- *update*—the update schedule;
- *restrictUpdate*—allows the product to completely disable updates, users might not want to update their products for several reasons as stated by design constraint 7;
- *restrictAccess*—allows users to disable remote control the product, vendors may enable this feature so that they can remotely connect to the product as stated by design constraint 7;
- *location*—the GPS location of the product.

For situations in which a product is stolen or altered, vendors have the option to disallow any incoming connections from that product.

#### 4.2.4. Application

The application object represents a set of parameters required to run a piece of software on a product. Each application is identified by a fully qualified domain name (FQDN). The application has a list of available software versions.

Software pieces are bundled into docker containers, meaning that they are shipped together with all the libraries required for the software to run. As these containers can become rather large, docker allows several versions of the same application to share the libraries component. This makes the deployment process more efficient as it enables differential updates in the way design constraints 3 and 9 state.

### 4.3. Details on the Repository

The repository is used to store the container images that the products download during the update process. Our repository implementation uses the official private docker registry [58] deployed using several kubernetes pods to which we have attached a persistent volume.

### 4.4. Details on the Deployer

Deployments are a link between an application, a version of an application, a product type, and a target. The technical implementation defines an extra set of run parameters that are specific to the application environment (e.g., configure container characteristics).

The deployer is the IoTWay component that handles the launch and management of applications on products. Besides managing the application containers, the deployer is responsible for collecting and reporting information about the product and, if needed, it creates a live link between the product and the vendor.

**Figure 4:**
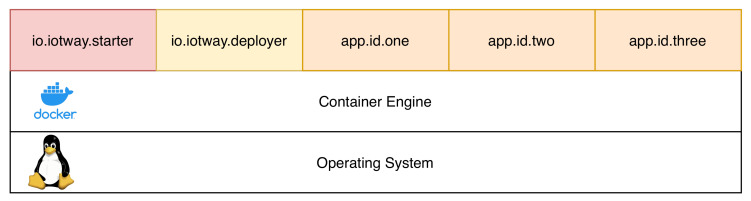
The IoTWay product software stack.

Figure 4 illustrates the product software stack. The product is running an operating system that can support containers. As defined by the design constraint 10, the IoTWay should integrate with any solution that the vendor already has, and any Linux operating system that allows containers should enable this easy integration.

On top of the operating system, a container engine is running that may be compiled as a static binary, thus it does not impose any restriction on what version of the Linux operating system it requires. Any container system whose kernel is capable of meeting the requirements for namespaces, cgroups, and overlayfs is compatible with the IoTWay model implementation.

All the applications that run on the product are packaged into containers and run by the container engine. The engine is the only piece of software running outside the container. This implies that the IoTWay deployer is itself a container. From a more detailed point of view, the deployer is configured as two containers: the starter and the actual deployer.

The purpose of the starter is to launch the deployer and make sure that it runs properly. One drawback of this system is that the starter is not updatable via the normal application update system. It can be updated only by a full system update.

The deployer, on the other hand, is updated in a similar manner to the applications. Once updated, the starter ensures that the new version of the deployer starts and keeps running. If the deployer fails to launch or crashes several times (18) in a row, the starter will classify this version as non-functional and it will revert the deployer to the previously known working version, while the server will be notified about the failure. With this approach, we can ensure reliable updates in the module underlying the application layer.

When asking for an update, each product will receive a list of deployments that are assigned to it (16). This consists of the deployments targeting the product’s cluster superposed with the deployments targeting that specific product. Further on, products will download all the containers specific to the versions of the applications in that list that are not already stored on the product. Next, the container-specific to the latest version of each application (19) will be run.

Storing several container versions for each application makes rollback fast and easy, as per design constraint 5.

In its implementation, the deployer relies on the following components:

- Setup—It is responsible for reading the product configurations and setting up the deployer;
- Uplink—It is responsible for connecting the product to the server. It uses the keys and (if available) the TPM to digitally sign exchange packets (28) and send them to the server;
- WebSocket—It is responsible for creating a permanent connection to the server. It is used by remote control components like *Shell* or *Remote*;
- Shell—It uses the *WebSocket* component and offers the ability to access the shell on the product remotely. Users are able to directly control the system on the product. This is not recommended for production environments;
- Remote—It uses the *WebSocket* component and offers the possibility to tunnel a network connection from the user to the product;
- Application Manager—It is responsible for managing the software that has to run on the product. In the scheduled update interval, it connects to the server via *Uplink* and downloads the new deployments manifest;
- Container Manager—It is responsible for managing the containers running on the product. Based on the input from the *Application Manager*, it downloads, starts, and stops containers.

**Figure 5:**
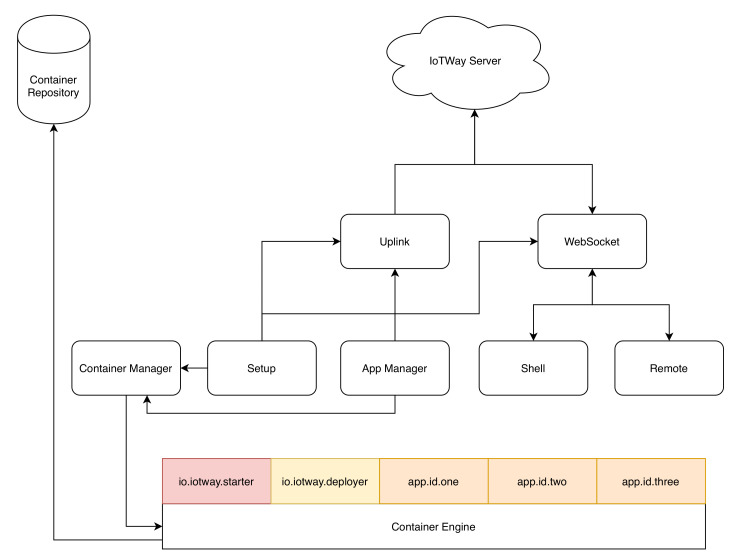
The IoTWay architecture components and how they communicate.

Figure 5 shows all the components and the relationships between them and their interaction with the container engine.

#### 4.4.1. Provisioning

Before a product is able to communicate with the server, it has to be provisioned. We defined two kinds of provisioning: *manual provisioning* and *self-provisioning*. While it first implies that the user manually adds the product to a cluster, *self-provisioning* allows products to register themselves with the server.

Products are shipped to the customers flashed with the specific provisioning information under the form of a provisioning file. The provisioning file is the implementation of the $\vec{m}$ vector (3) described in the mathematical model. It contains the cluster’s private key $k_{c}\left(\vec{m}\right)$ and a product’s private key $k_{p}\left(\vec{m}\right)$.

In our technical implementation, the provisioning file has a JSON format storing the necessary information.

The parameters $P_{a}$ are represented by several options related to the product interaction with the server:

- repository—the address of the repository where the containers are stored;
- server—the address of the server;
- shell—whether the device should allow remote access to it via a shell; this is not recommended for production products;
- access—whether the device should perform any communication with the server;
- update—whether the device should perform updates.

If possible, we recommend the placement of the keys ( $k_{c}\left(\vec{m}\right)$ and $k_{p}\left(\vec{m}\right)$) into the product’s TPM instead of the provisioning JSON file.

**Figure 6:**
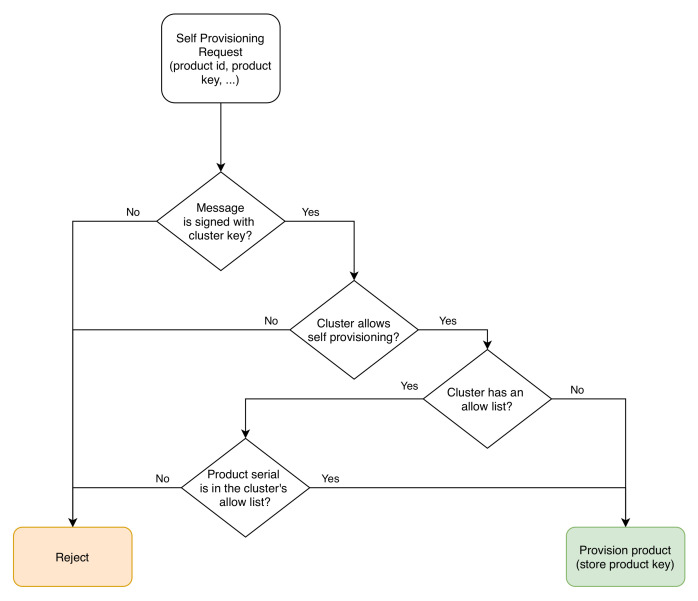
A flowchart showing the product self provisioning at the server.

The first time self-provisioned products connect to the server; they have to authenticate with the cluster’s private key $k_{c}\left(\vec{m}\right)$ and require to be provisioned (42). The server will authenticate the product with the public key and add the product to the cluster (44). In the technical implementation, products may be further filtered using a list of self-provisionable products (allow products) set in the cluster’s properties. Figure 6 describes the process.

After provisioning, the server will remember the product’s public key and will use it to further authenticate the product. The product will use its product private key $k_{c}$ to sign the exchange messages from now on ((28) and (43)).

#### 4.4.2. Scheduled Updates

To implement a solution suitable also for industrial usage, an important property consists of scheduled updates. This is important as for some products, such as vending machines, updates should not be performed during operating hours. This is in accordance with constraint 4.

This characteristic is implemented as a product property that stores a specific time frame when any updates should be performed. The deployer always checks this property’s value before querying the server for a new update. If the vendor does not specify a certain interval, a default interval is generated during the product provisioning phase.

### 4.5. Details on the Client

The client is an optional component that enables the interaction with the server and the products. In our implementation, it provides two management interfaces to the system: a WebUI developed with Vue.JS [59] and Bootstrap [60], and a command line interface developed in NodeJS.

**Figure 7:**
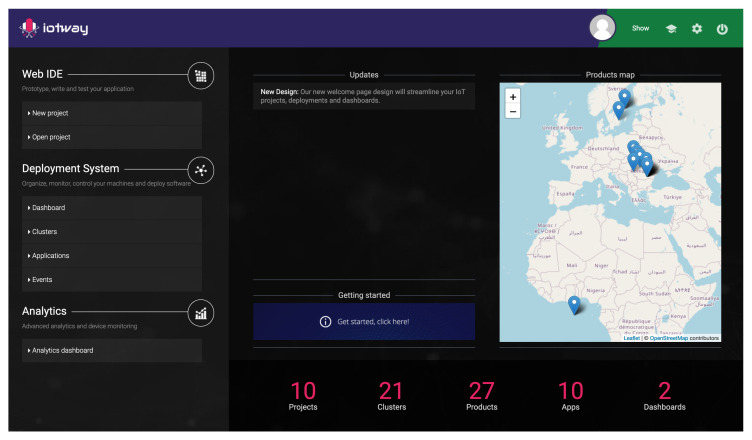
IoTWay dashboard.

From the WebUI, users are able to see a dashboard similar to the one in Figure 7, manage clusters, products, applications, deployments, and system events. For every product, users are able to view its functioning parameters, such as CPU usage, memory usage, application statuses, and even the display, according to design constraint 6.

The command line client allows users to perform the same tasks as with the WebUI, but through a shell. This is useful for writing development and deployment scripts and also for integrating our systems into other existing platforms. A novel feature provided by the command line client is the remote connections. This implies tunneling a network connection from a user’s computer to a device via the IoTWay server. The technology used for this is WebSockets. The client connects using a WebSocket to the server, while product does the same. Using this technology, we have successfully tunneled a Remote Framebuffer (RFB) [61] connection to the products, making development much easier.

### 4.6. Further Details on Server Design and Implementation

We have chosen HTTPS as the protocol for product server communication. The TLS layer of HTTPS allows the product to authenticate the server without any further implementation required on the server or product side. HTTPS implements the sign function from the exchange response packets ((39) and (45)).

**Remark** **4.** *For full HTTPS security, the server has to provide a valid verifiable CA certificate to the product.*

#### 4.6.1. Authentication

A special aspect of the communication between the server and the product is product authentication. First of all, as this is a production environment, authentication needs to be twofold: first, the product has to authenticate the server and, second, the server has to authenticate the product. The product uses an HTTPS link to the server and authenticates the server using the CA certificate authentication. As long as the public keys stored on the product are kept up to date, there should be no issue with this method.

The authentication of the product implemented differently. The product communicates with the server using a series of HTTPS POST messages that may be completely independent of each other and may be sent using different TCP connections. This series of message exchange is handled by the product’s *uplink*. As the HTTPS POST requests might be sent at very different time intervals and over several TCP and SSL connections, the link is susceptible to replay attacks [54]. Usually, this kind of attack may be stopped using timestamps, but, in our case, the involved devices might not have an RTC or might have a drifting clock. To prevent this, the connection uses a packet counter called *upFrame* and a *nonce*. This notion is inspired by the LoRaWAN protocol [62]. The product uses its private key, usually stored in the TPM, to digitally sign every message (packet) that it sends to the server.

The *uplink* may be defined in relation to two different product states: *unregistered* and *registered*. Before exchanging any relevant information, a product needs to register with the server to receive an authentication token and reset the packet counter. The server receives a register request and verifies the signature. If the signature is valid, it generates a unique, one-time-use, random token, and resets the packet counter to 0. It then sends the token to the product.

Upon receiving the registration response, the product is now in the *registered* state. From this point on, the product will increase the packet counter *upFrame* with each packet that it sends. The server will ignore any packets that have an *upFrame* lower than the frame counter it has stored in its database. When a new valid packet is received, the frame counter is set to that packet’s *upFrame* value.

**Figure 8:**
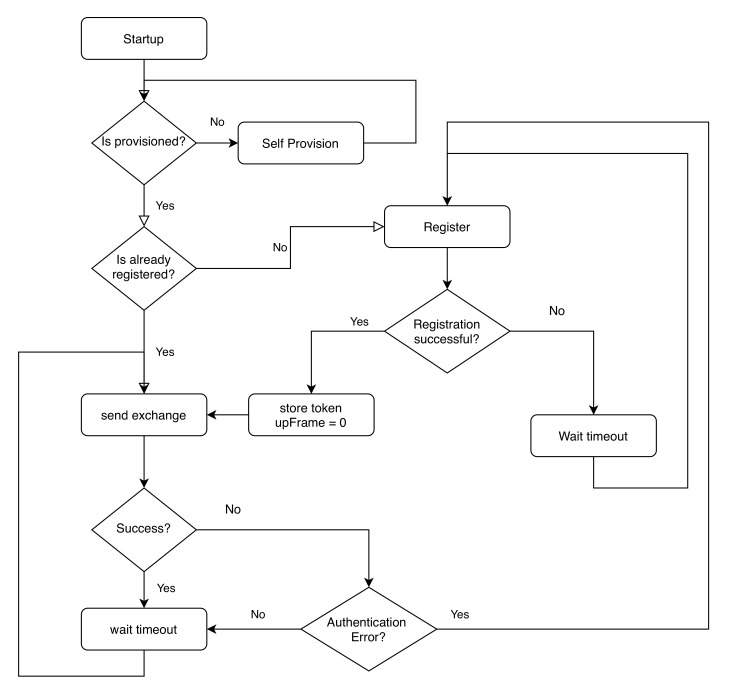
A flowchart showing the connection sequence between the product and the server.

Figure 8 describes the whole connection flow from the product’s point of view.

#### 4.6.2. Exchange

Due to the fact that products might have limited Internet bandwidth, HTTPS POST messages are sent to the server using an exchange schedule. The deployer’s *uplink* component will store all the requests from other components (e.g, shell, application manager) in a queue (22) and bundle them together in a periodical exchange packet with the server. Each packet *p* sent to the server is composed out of the *productId*, *nonce*, *upFrame*, *token*, and *payload*.

### 4.7. Security Policies

In order to ensure the security of the proposed system, we implemented several policies targeting multiple components of the infrastructure. With this, we aim to reduce the security surface attack and mitigate various security risks.

The first policy relies on authentication. Therefore, product and server authenticate each other using PKI mechanisms. Products authenticate the server using HTTPS messages, while the server authenticates the products using the cluster and the product key.

To ensure the security related to the device being exposed to external factors (all devices receive data from the cloud), we used both nonces and packet counters. This makes reply attacks hard to employ.

At the device level, isolation policies are implemented based on containers. All applications run in separate containers that do not have root privileges unless specifically required. This prevents applications from interfering with each other and accessing each other’s data. Real network interfaces are also hidden from applications unless necessary.

## 5. Discussion and Results

To assess the functionality of the model and technical implementation presented above, we implemented a management and update infrastructure on top of which smart soda dispenser machines were deployed. To evaluate the efficiency of the model, at each layer of the IoT stack, we employed multiple different technologies with a twofold purpose. First, we aim to measure the impact the update process has on the performance of the rest of the components (e.g., increased energy consumption, high network load that leads to unstable connections). Secondly, the target is to build a deployment infrastructure that can be integrated with various heterogeneous systems. Therefore, the target is to obtain a general, stable, secure, and efficient implementation of the update model presented in the previous sections.

The use-case consists of multiple soda dispenser machines connected to the cloud with the purpose of uploading status and consumption data. All machines integrate various sensors measuring the water filter status, the quantity of disposed beverage, the machine temperature and energy consumption. The users interact with the dispenser via a touchscreen that displays selection buttons for the beverages and a start/stop button to control the liquid flow. Furthermore, the vendors have access to a management interface where they can manipulate the dispensers, view their status (e.g., connected/disconnected, running/not running, expired water filter) and update the software.

### 5.1. Technologies Used

In building up the proposed use-case, we tried several approaches with the aim of identifying the most suitable solutions. Further on, we describe the technologies we used together with the advantages and the disadvantages we identified.

#### 5.1.1. Hardware

The hardware integrated into the dispensing machines consists of an embedded computer that is connected to electromechanical relays controlling the liquid pumps and to a smart filter that measures the dispensed liquid quantity. For the embedded computers, we decided to work with two of the most popular platforms: Raspberry Pi [63] and BeagleBone Black [64].

The Raspberry Pi is one of the most used prototyping embedded computers and is very robust and resilient to short circuits and current spikes. Although not initially designed for industrial use, the Raspberry Pi has an industrial version. Vendors provide this version in robust encases exposing specific industry-standard connectors. As a result, many commercial and industrial IoT applications consist of Raspberry Pi devices [65].

The BeagleBone board, on the other hand, is easier to integrate into other devices as it is open hardware. The BeagleBone schematics are public and any producer can adapt it to their requirements and build their own device. In regard to the specific BeagleBone Black device that we used, it has reduced capabilities compared to the Raspberry Pi, which proved to be unsuitable for this use-case. This result is dependent on the software’s characteristics, which is detailed in Section 5.4.

#### 5.1.2. Software

For both of the embedded computers, Raspberry Pi and BeagleBone Black, we used the official operating system distribution promoted by the hardware producers, both Debian-based. Both images are the stripped-down versions, without the graphical interface.

To implement the containers on top of which the applications run, we used two of the most common technologies: Docker [44] and Balena [8]. Both of them were statically compiled, resulting in one single binary. While Balena was specifically designed for embedded devices, we experienced (at the time of implementation, 2019) several container engine crashes. Docker, on the other hand, proved to be very stable, so the final product was shipped with Docker.

### 5.2. Network Connection

An important parameter in the implementation is the device network connection. As the software transfer is made from the cloud using the HTTPS protocol, an Internet connection is required. In the presented use case, we used Ethernet, Wi-Fi, and 4G to connect the dispensers to the cloud. This enabled us to test the deployment infrastructure over a stable network connection (Ethernet) but also over connections that had a high rate of packet loss (Wi-Fi and 4G).

The Ethernet connection supported transfers of 100 Mb/s with no transmission errors. The 4G connection for some of the devices had 10% packet loss. In several cases, the Wi-Fi gateway was placed in a sub-optimal position by the commercial partner that handled the physical deployment, leading to poor signal quality and resulting in an approximate rate of 30% packet loss. This enabled us to test the system’s efficiency for devices deployed in remote areas having limited network access.

### 5.3. Cloud Infrastructure

We have designed our implementation using kuberenetes [66] versions 1.7 and 1.12 clusters. The server is a collection of REST micro-services running on several pods.

A MongoDB [67] distributed database has been used for persistent data storage. We have used MongoDB Atlas [68] and Azure DocumentDB [69]. While both of them had a very good response time (less than 1 ms), Document DB proved to be very expensive as it is charged per request. For about 50 devices with under normal functioning, pricing went up to around $2000/month. On the other hand, MongoDB Atlas proved to be slower in response then Azure Document, having a response time of around 5 ms. Table 2 shows a comparison of the two.

As speed for Azure DocumentDB was very good but it was charged per query, we discovered that most of the queries were due to users logging in and performing actions. To optimize, we used a Redis [70] High Availability cluster for cashing data. This reduced the cost to around $100/month and improved the query speed by around 70%.

**Remark** **5.** *MongoDB Atlas is charging by data size, not per request. The $50 price was offering 10 GB of storage out of which IoTWay used less than 500 MB.*

We used two cloud providers to deploy the kuberenetes cluster: Azure AKS (preview at the time) [71] and Amazon EKS. While Azure AKS was easier to set up, the kubernetes control plane being fully managed by Azure, we had a lot of issues with pods being stuck in *Terminating* status and nodes sometimes disconnected. As Azure had no SLA at the time, we had to switch to another cloud provider and we chose Amazon Web Services.

**Figure 9:**
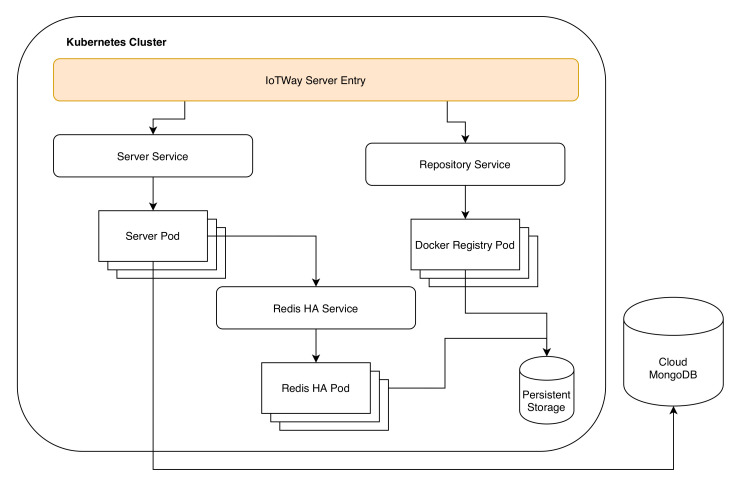
IoTWay server infrastructure.

The Amazon EKS setup was not that straightforward; we had to provision the control plane nodes more or less manually. After that, everything went pretty smooth. We still experienced some pods being stuck in Terminating, but much less often than in AKS. Amazon AKS did offer an SLA at the time, issues were quickly solved. The server infrastructure is described in Figure 9.

### 5.4. Deployed Software

During the implementation of this model, we have run several applications on products ranging from simple data acquiring software to applications that have a display and interact with the users.

The largest deployment of products that we have done is around 100 soda dispenser machines, running in Romania, India, and the United States. The software running on the products was designed as an electron [72] application running on top of an Xorg Server [73].

As Table 3 points out, machines built on top of the BeagleBone Black were not able to properly run the soda dispenser application. The Raspberry Pi, having four cores, was able to perfectly run the software and performance improved significantly when GPU render was active. The load average is computed as the average CPU usage (in %) ore a time span of 10 min. We ran the same software on the three devices, and as it can be seen from the numbers, the average load greatly decreases when using the GPU render. The data confirm what we suspected: that most of the CPU and memory load was the result of the UI software render.

Due to the high load on the BeagleBone Black, we experienced a high amount of network packet loss and disconnects. Even in these conditions, we were able to eventually successfully update the machines, the system being able to recover the machines from several update failures.

### 5.5. Updates Performances

To evaluate the model’s performance, we measured the size of the first deployment and updates. These differ as the platform is designed to support differential updates. In this context, during the first iteration, the first deployment image size was around 1.2 GB, while the updates ranged between 200 MB and 300 MB. To optimize, we decreased the base container size by creating a more efficient built system and identifying and dropping unnecessary files created during the built process. In addition, we reduced the number of messages being exchanged, resulting in an initial deployment size of 500 MB and updates size ranging between 50 MB and 100 MB.

Following the optimization, the update retry rate decreased from 20% to 5% as most of the failed updates were due to the unreliable network connection. Reducing the traffic resulted in a higher update success rate.

When considering the update time, the initial deployment time decreased from 1 h to 20–35 min, while the update time decreased from 10–15 min to 5 min.

These results were obtained using a Raspberry Pi device (Table 4). For the BeagleBone, the update retry rate and update time are 30% higher due to the hardware limitations.

Once update performances were improved, we used the system for the whole development process of the soda dispenser machines. In total, for this use-case, we performed 133 software releases on 100 soda dispensing machines (Table 5). Out of the total number of updates, 20 machines underwent complete failures, most of them due to faulty hardware storage (SD card failures). In addition, due to the hardware limitations of the BeagleBone Black devices, 30% of the total software deployments failed. Most of the failures resulted from faulty disk writes and network packet losses. However, the system maintained stability as the update infrastructure automatically rolled back all non-functioning devices to the last working applications version.

Overall, we consider the use-case as a successful implementation of the update system in a commercial production environment.

### 5.6. Comparison of the Presented Model with Other Models

The solution proposed in the paper aims to address all the major aspects related to IoT updates in sensor networks by providing a mathematical model to characterize a generic OTA update mechanism. This comes in response to a lack of generic updates platform that we identified when analyzing other solutions in both research and commercial literature. Therefore, our aim is to propose a general model that can be implemented and adapted to any specific use-case.

However, when designing the proposed model, we took into account other existing solutions and their proposed approaches. Furthermore, the mathematical model is presented in direct relation to a technical implementation meant to validate it, which can be compared with other existing platforms. As the model aims to address commercial use-cases, a comparison with the platforms identified in Section 2.2.4 is appropriate.

Besides the characteristic generality of the model we propose, the platform is also built on top of open technologies and is designed to be easily integrated with any third-party services and deployed on users’ premises. In contrast, most of the existing solutions are provided as software as a service, which forces the users to integrate with a specific account and application store such as Ubuntu One in the case of Ubuntu Core [6], or Android Console in the case of Android Things [5].

An important aspect about IoT updates is the capability to recover after a failed update, which in some systems [5,7] relies on A/B partitions. As one of the partitions is active, the other is used for the update and only if the process succeeds does the latter become the active partition. However, this requires the system to reboot for the new version to be in place. In the case of the proposed solution, the container mechanism ensures the updates are made in a robust manner, without the need to reboot the system. This is similar to the Balena [8] platform. Furthermore, as all applications run on top of containers, application security and process isolation are enforced, a more reliable security mechanism than the ones enforced by platforms relying on permissions [5,6].

In terms of performance, we compared IoTWay, with Balena as we identified this to be implementation most similar. To this end, we performed application deployment on both Raspberry Pi and BeagleBone Black devices using both the proposed platform and Balena, the latter resulting in an increased number of failures. These results are due to the technical implementation, which in our case relies on standard docker containers, while Balena uses a custom version of the same container. Therefore, at the time of our tests, the Balena containers proved to be unstable for ARM devices, resulting in arbitrary failures (Table 6).

The main difference in the proposed model and the Balena platform consists in a larger number of unrecovered updates and devices having unrecoverable failures unrelated with any update.

### 5.7. Limitations and Future Improvements

While proving to be an efficient application updates solution, the proposed model has its limitations related to kernel updates. The mathematical model was designed to efficiently support robust, fail-safe application updates, but does not handle the update process of the underlying software (kernel updates). As an important future improvement, the mathematical model and the corresponding technical implementation need to be adapted to support both application and kernel updates.

Another limitation, which is solely related to the technical implementation of the model, consists of the container technology used. Currently, the IoTWay platform is compatible with docker container only, as it can be improved to work with other technologies such as snap [74], flatpack [75], or rocket [76].

Another important future improvement is to adapt the model for industrial usage. This requires more focus on the robustness of the model and on making it compliant with necessary certifications and security policies.

## 6. Conclusions

This paper presents a novel model for a remote software update system dedicated to sensor-based IoT infrastructures, backed-up by an in-depth field overview and a mathematical model, and finally validated through a real-world deployment of a commercial IoT solution.

The remote software deployment and update architecture proposed for sensors infrastructures are based on a mathematical model that grants robustness to the approach, while also empowering other researchers and commercial vendors and system integrators to explore and deploy similar infrastructures. This is built on top of an in-depth domain overview that offers other developers a detailed synopsis that can serve as a ground base for further model extensions.

For validation, we used the model to implement a real-world medium-size commercial IoT deployment—for a commercial partner that required frequent updates for the software running on multiple soda dispenser machines. The machines were deployed in three geographical locations across Romania, India, and the United States.

The deployment covered 100 soda dispensers with integrated sensors and smart controllers that run the software deployed through our platform. These underwent 133 remote software updates in a 250 day time-frame, with 80% of the machines running uninterrupted, and 20% suffering complete failure due to hardware faults. Out of the total 13,300 software deployments, 30% failed, resulting in the automatic rollback of the system. This ensured that all the connected devices continued to function, resulting in 100% reliability of the implemented use-case.

Thus, our current work provides both researchers and commercial developers with a robust model that will enable fast, reliable, and secure remote software updates—allowing for agile development, fast security update response, and reduced deployment cost for isolated locations.

Our current work covered a remote software deployment and update system aimed at commercial sensor-based IoT deployments. For industrial IoT applications, we aim to further develop our model for enhanced robustness and certification compliance, and test it out in an industrial scenario.

## Figures and Tables

**Table 1:** Commercial IoT deployment solutions and their properties.

Solution	Update Format	Update Type	Connection	Account	Security
Android Things	APK—Similar to Android applications	Differential; Full-system—relies on a dual partition mechanism to avoid lockout	Passive—a service asks every few hours for available updates	Requires Android Console account	Based on permissions
Ubuntu Core	Snap package	Transactional updates; Single application or kernel can be updated at once	Passive	Requires Ubuntu One account	Based on permissions— snap connectors
Mender	Mender Artifact	Differential; Full system or application—dual partition mechanism to avoid lockout	Passive—manually configured polling interval	Can be installed locally	Each application handles its isolation
Balena	Docker image	Full-system and differential updates	Active—devices are notified when a new version is available	Can be installed locally or sign up with Balena cloud	Based on containers

**Table 2:** Pricing and average query speed for MongoDB used for 100 devices and 3 users.

Database	Pricing (USD/Month)	Speed/Query
MongoDB Atlas	50	5 ms
Azure DocumentDB	2000	1 ms
Azure DocumentDB (with redis cache)	100	0.3 ms

**Table 3:** Machine performance.

Platform	CPU Speed	RAM	Avg Load	Avg RAM Load
BeagleBone Black	1.0 GHz	512 MB	150%	100%
Raspberry Pi 3 (no GPU driver)	4 x 1.2 GHz	1 GB	40%	60%
Raspberry Pi 3 (GPU driver)	4 x 1.2 GHz	1 GB	10%	60%

**Table 4:** Update performances.

	Initial Deployment Size	Update Size	Update Retry Rate	Initial Deployment Time	Update Time
Initial	1.5 GB	500 MB	20%	1 h	10–15 min
Optimized	200–300 MB	50–100 MB	5%	35–20 min	5 min

**Table 5:** Update numbers.

Platform	Devices	Updates	Avg. Recovered Devices/Update	Avg. Unrecovered Devices/Updates	Devices with Other Failures
BeagleBone Black	80	133	25	2	20
Raspberry Pi 3	30	133	3	0	0

**Table 6:** Update performance comparison between IoTWay and Balena.

Platform	Devices	Updates	Avg. Recovered Devices/Update	Avg. Unrecovered Devices/Updates	Devices with Other Failures
IoTWay	110	133	19	2	20
Balena	110	133	17	10	40

## Abbreviations

The following abbreviations are used in this manuscript:

API	Application Programming Interface
ARM	Advanced RISC Machine
CA	Certification Authority
CPU	Central Processing Unit
CoAP	Constrained Application Protocol
DAPS	Double Authentication Preventing Signature
FQDN	Fully Qualified Domain Name
IoT	Internet of Things
IIoT	Industrial Internet of Things
JSON	JavaScript Object Notation
MQTT	Message Queuing Telemetry Transport
OABS	Outsourced Attribute-Based Signature
OTA	Over The Air
PKI	Public Key Infrastructure
REST	Representational State Transfer
RFB	Remote Framebuffer
TLS	Transport Layer Security
SSH	Secure Shell
SGX	Software Gurad Extension
TPM	Trusted Platform Module
VNC	Virtual Network Computing
